# Preparing healthcare facilities in sub-Saharan Africa for future outbreaks: insights from a multi-country digital self-assessment of COVID-19 preparedness

**DOI:** 10.1186/s12913-024-10761-2

**Published:** 2024-02-28

**Authors:** Gloria P. Gómez-Pérez, Aafke E. de Graaff, John T. Dekker, Bonifacia B. Agyei, Ibironke Dada, Emmanuel Milimo, Marilyn S. Ommeh, Peter Risha, Tobias F. Rinke de Wit, Nicole Spieker

**Affiliations:** 1 PharmAccess Foundation, Amsterdam, The Netherlands; 2 Amsterdam Institute for Global Health and Development, University of Amsterdam, Amsterdam, The Netherlands; 3 PharmAccess Ghana, Accra, Ghana; 4 PharmAccess Nigeria, Lagos, Nigeria; 5 PharmAccess Kenya, Nairobi, Kenya; 6 PharmAccess Tanzania, Dar es Salaam, Tanzania

**Keywords:** COVID-19, Africa, Digital tools, Epidemic preparedness, Quality improvement

## Abstract

**Background:**

Despite previous experience with epidemics, African healthcare systems were inadequately prepared and substantially impacted by the coronavirus disease 2019 (COVID-19) pandemic. Limited information about the level of COVID-19 preparedness of healthcare facilities in Africa hampers policy decision-making to fight future outbreaks in the region, while maintaining essential healthcare services running.

**Methods:**

Between May–November 2020, we performed a survey study with SafeCare4Covid − a free digital self-assessment application − to evaluate the COVID-19 preparedness of healthcare facilities in Africa following World Health Organization guidelines. The tool assessed (i) COVID-19-related capabilities with 31 questions; and (ii) availability of essential medical supplies with a 23-supplies checklist. Tailored quality improvement plans were provided after assessments. Information about facilities’ location, type, and ownership was also collected.

**Results:**

Four hundred seventy-one facilities in 11 African countries completed the capability assessment; 412 also completed the supplies checklist. The average capability score on a scale of 0–100 (*n*=471) was 58.0 (interquartile range 40.0–76.0), and the average supplies score (*n*=412) was 61.6 (39.0–83.0). Both scores were significantly lower in rural (capability score, mean 53.6 [95%CI:50.3–57.0]/supplies score, 59.1 [55.5–62.8]) versus urban facilities (capability score, 65.2 [61.7–68.7]/supplies score, 70.7 [67.2–74.1]) (*P*<0.0001 for both comparisons). Likewise, lower scores were found for public versus private clinics, and for primary healthcare centres versus hospitals. Guidelines for triage and isolation, clinical management of COVID-19, staff mental support, and contact tracing forms were largely missing. Handwashing stations were partially equipped in 33% of facilities. The most missing medical supply was COVID-19 specimen collection material (71%), while 43% of facilities did not have N95/FFP2 respirators and 19% lacked medical masks.

**Conclusions:**

A large proportion of public and private African facilities providing basic healthcare in rural areas, lacked fundamental COVID-19-related capabilities and life-saving personal protective equipment. Decentralization of epidemic preparedness efforts in these settings is warranted to protect healthcare workers and patients alike in future epidemics. Digital tools are of great value to timely measure and improve epidemic preparedness of healthcare facilities, inform decision-making, create a more stakeholder-broad approach and increase health-system resilience for future disease outbreaks.

**Supplementary Information:**

The online version contains supplementary material available at 10.1186/s12913-024-10761-2.

## Background

In December 2019, the first case of a novel coronavirus (severe acute respiratory syndrome coronavirus 2 [SARS-CoV-2]), was officially reported in Wuhan, China [1]. Soon after, a pandemic of coronavirus disease 2019 (COVID-19) emerged, reaching Africa in February 2020 [2], and spreading rapidly to as many as 52 African countries by April the same year [3]. As of August 3, 2023, there were officially 12,216,748 COVID-19 cases and 256,542 related deaths reported in this region [4]. These statistics are likely an underestimation due to low COVID-19 testing capacity [5] and unreliable mortality data in most African countries [6]. In a post-mortem study in Zambia, it was found that most people with COVID-19 (51/70, 73%) died in the community, with none of them having a COVID-19 test before death [7]. Moreover, due to the high prevalence of mild or asymptomatic COVID-19 infections in Africa, many people did not get tested. Overall, in the Africa region only 1.4% of SARS-CoV-2 infections were reported [8]. Therefore, COVID-19 is not less prevalent in Africa than in other parts of the world but is less tested/reported and shows different epidemiologic characteristics.

At the population level, containment measures developed by/for industrialized countries were difficult to adhere to in Africa due to high rates of informal employment. Work-related mobility during the pandemic was more prevalent in poor-income settings, with higher poverty inducing a faster spread of the virus [9]. At a healthcare facility level, management of airborne diseases require containment protocols (infection prevention and control [IPC]) that are difficult to implement, i.e., hand hygiene, physical distance, and access and correct use of personal protective equipment (PPE), as recommended by the World Health Organization (WHO) [10]. Of concern, it was previously found in Tanzania that only 6.9% among 220 private outpatient facilities complied with the most important basic recommendation of the WHO to fight the COVID-19 pandemic: hand washing [11]. The absence of this and other life-saving infrastructure and supplies, such as basic PPEs, were responsible for the high infections rates among healthcare workers in Africa [12, 13] that raised by 203% from May to July 2020 [14].

In Africa, as in other regions with fragile healthcare systems and lack of epidemic preparedness, pandemic-related disruptions of healthcare services have seriously increased the inequalities of access to care with devastating consequences. The lack of surge capacity, defined as “the ability to obtain adequate staff, supplies and equipment, structures and systems to provide sufficient care to meet immediate needs of an influx of patients following a large-scale incident or disaster” [15], meant that human and economic resources needed to be allocated from essential healthcare services to COVID-19 containment efforts. For instance, COVID-19 caused the most widespread and largest global disruption of routine immunization in 30 years, leaving millions of children under-vaccinated or unvaccinated [16, 17], resulting in several outbreaks of vaccine preventable diseases in 24 countries in the African region, including the return of wild polio virus [18]. Similarly, there were significant pandemic-related disruptions in HIV, TB, malaria services, and maternal and chronic non-communicable diseases care [19–22]. It will take many years to reach the pre-pandemic progress of routine immunization programs and control of these diseases in the region.

Undoubtedly, outbreaks of emerging infectious diseases (EIDs) will continue occurring, as it has been shown by the recent outbreaks of zoonotic diseases: Monkeypox [23], Sudan Virus Disease (Ebola) [24], and the re-emerge of COVID-19 cases in China in 2022 [25]. The likelihood of EIDs is considerably higher in countries located in forested tropical regions with high wildlife biodiversity and experiencing land-use changes [26]. Worryingly, the reporting effort and epidemic preparedness in these countries is low [27], compared with regions with the highest levels of national epidemic preparedness located in Europe and North America [28] where the likelihood of EIDs is lower. This represents a significant threat to global health since the drivers of disease emergence are likely to continue and intensify. Action needs to be taken to significantly improve the epidemic preparedness in the countries with high risk of disease emergence and fragile healthcare systems to detect and control outbreaks promptly.

To improve epidemic preparedness, metrics are crucial to assess resilience to infectious diseases outbreaks [29]. However, in Africa there is scarcity of epidemic preparedness data from healthcare facilities that could guide implementations aiming at closing the gaps in the emergency response observed during COVID-19. Importantly, the healthcare provider perspective is rarely captured in such assessments, while doctors, nurses and other healthcare personnel are the ones at the front line in the response to such public health emergencies.

During the COVID-19 pandemic, witnessing first-hand the surge of fears among healthcare providers in Africa and the lack of information from the local authorities about COVID-19 containment measures and protocols, we developed in May 2020 ‘SafeCare4Covid’. This was a free and globally accessible digital self-assessment tool based on WHO guidelines and following a quality improvement methodology, aimed at helping healthcare facilities in low-middle income countries (LMICs) to get prepared to safely attend and care for COVID-19 patients. The tool provided real time information on preparedness to stakeholders in the health sector to make evidence-based decisions on how to allocate resources and patients [30]. In this paper we describe the data obtained by SafeCare4Covid on the level of COVID-19 preparedness and availability of supplies in 471 facilities across 11 African countries from May to November 2020, that could guide epidemic preparedness efforts in these countries for future outbreaks.

## Methods

### Study design and setting

We performed an observational study using SafeCare4Covid, a free, globally available digital self-assessment application, following COVID-19-related WHO guidelines and a quality improvement methodology, to assess the COVID-19 preparedness of primary, secondary, and tertiary healthcare facilities in Africa.

### SafeCare4Covid application

The SafeCare4Covid App was developed by PharmAccess, an international non-governmental organization (NGO), as part of its health systems approach to help African countries mitigate the effects of the COVID-19 pandemic. After developing and finetuning its content with staff from PharmAccess in Nigeria, Kenya, Ghana, and Tanzania, the SafeCare4Covid App was rolled out and used to assess healthcare facilities on their preparedness level against COVID-19, and communicate reliable, evidence-based data to them through information boxes and educational posters in English, French, Spanish and Swahili. Facility staff could access the App, self-evaluate against the questions, and upload the information digitally. SafeCare4Covid subsequently provided through each assessment a report and a customized quality improvement plan (QIP). Afterwards, the (meta-) data collected was shared through comprehensive dashboards with public and private sector stakeholders and donors for informed decision making [30]. SafeCare4Covid is an adaptation of the SafeCare methodology to improve quality of care [31]. SafeCare standards are accredited by the International Society for Quality in Health Care External Evaluation Association (IEEA), former International Society for Quality Health Care (ISQua) and is adapted to facilities in LMICs. Informed consent is provided by accepting the SafeCare4Covid Terms and Conditions that authorize to share data via dashboards with stakeholders, and to analyse and publish data with public health relevance in an anonymized manner. Facilities are encouraged to implement the changes advised by the QIP and to repeat the self-assessment at a later timepoint to track progress.

### Data security and management

SafeCare4Covid database is cloud-based and used to store information collected by the tool following General Data Protection Regulation (GDPR) principles according to EU-Regulation 2016/679 and the Organization for Economic Co-operation and Development (OECD) Privacy Principles.

### Participating facilities

Healthcare facilities of all types, locations, and ownerships were invited to use the tool. Although the application is globally available, the priority targets were healthcare centres in LMICs. Survey data was entered by staff responsible for quality which are usually nurses or medical doctors, and in consultation with other members of staff. This analysis is limited to the facilities located in Africa that have completed the SafeCare4Covid self-assessment in the period of May 27–November 25, 2020. Some facilities performed multiple assessments on the same day. When this happened, only the first data entries were included in this analysis. No facilities in this cohort repeated the self-assessments on dates different from the first assessment date. All users agreed with the Terms & Conditions before proceeding with the self-assessment.

### SafeCare4Covid assessment of capabilities and supplies checklist

The application includes three sets of questions that take an average of 30 min to respond: (i) *Demographic data*, the questions in this section collect information about location (rural, urban), type of facility (outpatient clinic, general hospital, specialized hospital), ownership (public, private for- profit, private non-profit, and faith-based), among others (Table 1). We assumed the providers classified their location as urban or rural according to standard definitions [32].

**Table 1:** Demographic characteristics of healthcare facilities per country

		Location			Type of facility				Ownership				
Country	#Facilities	Rural	Urban	N/P^a^	Outpatient Clinic	General Hospital	Specialized Hospital	N/P	Faith based	Private for profit	Private non-profit	Public	N/P
Kenya	190	66	41	83	64	66	–	60	11	49	23	47	60
Tanzania	114	79	24	11	67	41	1	5	27	2	6	74	5
Ghana	95	50	39	6	34	51	5	5	60	13	9	8	5
Nigeria	35	2	17	16	10	11	9	5	–	16	7	7	5
Namibia	21	5	14	2	16	1	2	2	–	18	–	1	2
Uganda	10	–	10	–	5	5	–	–	–	8	2	–	–
Ethiopia	2	–	2	–	2	–	–	–	–	–	2	–	–
Burundi	1	–	1	–	1	–	–	–	–	1	–	–	–
DRC	1	–	1	–	1	–	–	–	–	–	1	–	–
Malawi	1	–	1	–	–	1	–	–	1	–	–	–	–
Mozambique	1	1	–	–	1	–	–	–	–	–	1	–	–
Total	471	203	150	118	201	176	17	77	99	107	51	137	77
Proportions	100%	43%	32%	25%	43%	37%	4%	16%	21%	23%	11%	29%	16%

*Abbreviation:* *N/P *not provided

^a^Providing demographic information as location, type, and ownership of facility, is optional in the SafeCare4Covid application

If a facility did not respond to some of the demographic questions, here we report this as not provided (N/P);

(ii) *Supplies checklist*, this set of questions, which is based on WHO COVID-19 related recommendations, verifies the availability of 23 essential medical supplies (Supplementary Table 1), including PPEs. Responding to this section is optional. The users are given the possibility to respond ‘yes’, ‘no’, or ‘not applicable’ (NA) if they consider a supply is too advanced for their type of facility. If this section is completed by the user, SafeCare4Covid calculates a ‘Supplies score’ from 0 to 100 following a predetermined formula (Supplementary Methods); (iii) *Self-assessment of capabilities*, this section includes 31 questions (shown in the Results section) based on WHO COVID-19-related recommendations to healthcare providers and SafeCare methodology criteria. These questions cover four categories of COVID-19 preparedness: clinical management (10 questions [q]), infrastructure and supplies (12q), capacity building (4q), and IPC (5q). It is mandatory to answer all 31 questions to complete and submit the SafeCare4Covid evaluation. In this section the user can give three possible answers classified by the tool as non-compliant (NC), partial compliant (PC), fully compliant (FC), and NA. According to the answers provided, SafeCare4Covid calculates a ‘Capabilities score’ from 0 to 100 following a predetermined formula (Supplementary Methods).

The supplies and capabilities scores and the QIP are generated in real time. The QIP can be downloaded directly on a mobile phone, tablet, or computer, or can be sent by email. The QIP is adapted to LMICs to maximize the likelihood of the implementation of recommendations. For instance, if under the infrastructure and supplies evaluation a facility does not comply (NC) with access to running water, the QIP proposes as possible solution the use of Veronika buckets [33], that are of low cost and easy to implement.

### Statistical analysis

Facilities were included in the analysis if located in Africa, and with completed self-assessment questions. We analysed the level of compliance (proportions) with the 31 self-assessment questions/capability criteria classified in four levels: NC, PC, FC, and NA; and the availability status of 23 medical supplies in the healthcare centres that completed the ‘Supplies checklist’ answered as: Yes, No, and NA. In addition, we assessed differences in supplies and capabilities scores depending on the location, type, and ownership of the facilities. The scores’ data were not normally distributed. Hence, we performed Wilcoxon-Mann-Whitney rank-sum tests for two-group comparisons, and Kruskal-Wallis tests for multiple group comparisons, with significant level α = 0.05. When the Kruskal-Wallis test was rejected, a post hoc Dunn’s test was performed for pairwise multiple comparisons with Bonferroni adjustment, with family-wise error rate (FWER) = 0.05, and a significant level α = 0.05/total number of tests. Data was analysed in Stata/SE 16, and graphs were generated in Microsoft® Excel® for Microsoft 365 for MSO and Stata/SE 16.

## Results

We analysed SafeCare4Covid data from 471 facilities located in 11 African countries (Table 1) of which 313/471 (66.5%) facilities were participating in the quality improvement SafeCare programme, among which 272/412 (66.0%) completed the supplies checklist. The majority of facilities was located in Kenya (190/471, 40%), Tanzania (114/471, 24%) and Ghana (95/471, 20%). In addition, most of the facilities were of rural location (203/471, 43%), outpatient clinics (201/471, 43%), and of public ownership (137/471, 29%). Among these facilities, 412/471 (87.5%) completed the ‘Supplies checklist’ (Supplementary Table 1).

**Fig. 1:**
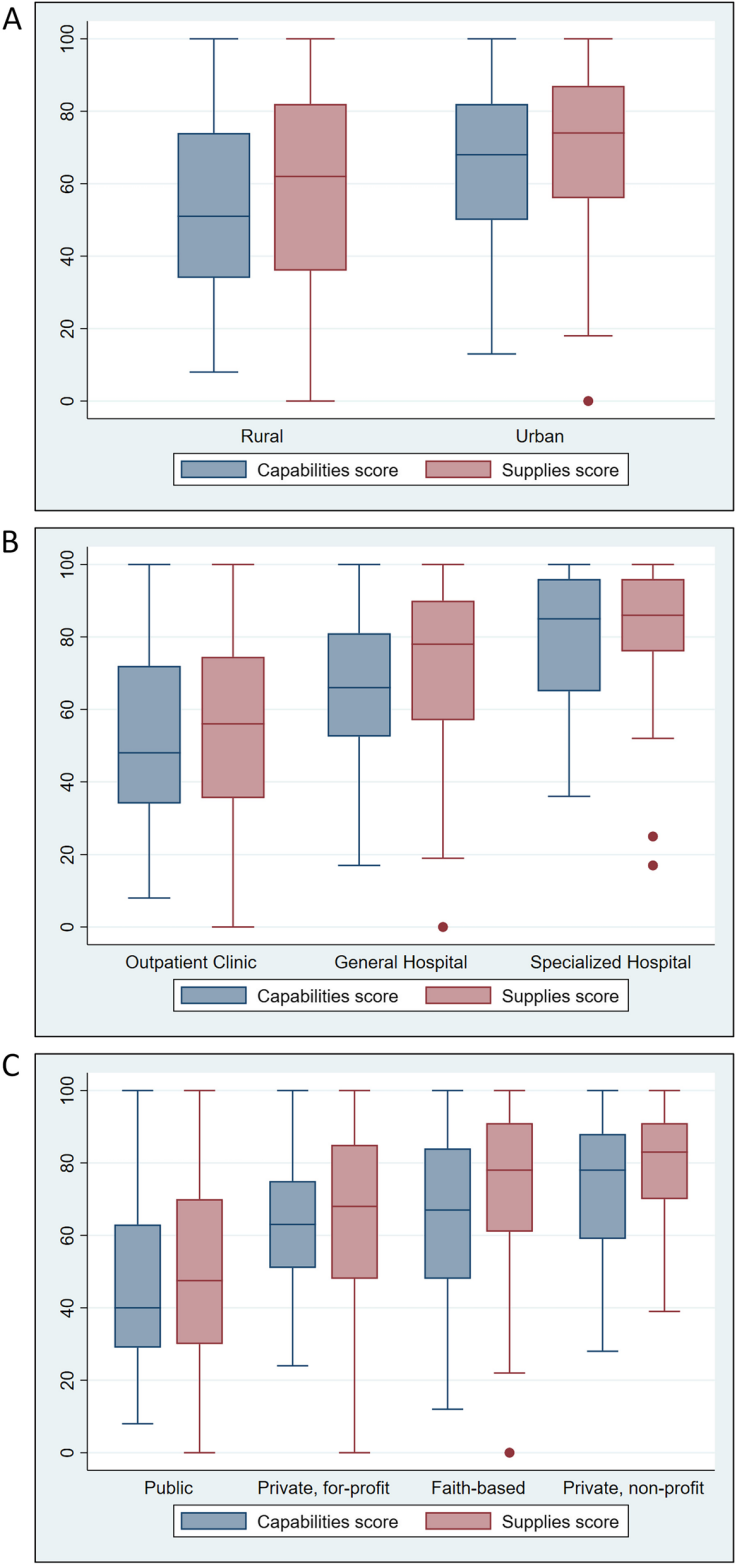
Capabilities and supplies scores by healthcare facility location, type, and ownership.  This figure shows SafeCare4Covid score distributions by location of healthcare facilities (**A**), type of facility (**B**), and ownership of facilities (**C**). Box plots visually represent the statistical comparisons described for this group of facilities in the Results section. They show the lower quartile (Q1), the median (Q2, line plotted inside the box), the upper quartile (Q3), and the interquartile range (IQR = Q3 - Q1) of the capabilities and supplies scores. The whiskers show the most extreme data points that are ≤ 1.5 times the IQR from the lower or upper quartile. Dots show the outliers (> 1.5 times the IQR). Since filling in demographic information is optional in the SafeCare4Covid App, not all the locations, types, and ownerships of the 471 facilities are known. Detailed samples sizes for the different groups compared here are described in Table 1

The average supplies score in this group of healthcare centres (*n* = 412) was 61.6 (interquartile range 39.0 to 83.0), and the average capability score (*n* = 471) was 58.0 (40.0 to 76.0). Facilities already in the SafeCare program had higher supplies scores (63.3 [43 to 83]) than facilities not in the SafeCare program (58.3 [39 to 83]), and also higher capability scores (58.6 [40 to 78] versus 56.9 [41 to 75]), but the differences were not significant (*P* = 0.10/*P* = 0.69, respectively). Regarding the location of healthcare centres, rural facilities had significantly lower supplies scores (mean 59.1 [95% CI 55.5 to 62.8]) than urban facilities (70.7 [67.2 to 74.1]), *P* < 0.001; and also significantly lower capability scores (53.6 [50.3 to 57.0]) than urban facilities (65.2 [61.7 to 68.7]), *P* < 0.001 (Fig. 1). Concerning the type of facilities, outpatient clinics had significantly lower supplies scores (55.5 [52.1 to 58.9]) than general hospitals (71.4 [68.0 to 74.7]), and specialized hospitals (77.5 [64.6 to 90.4]) (*P* < 0.001 for both comparisons; adjusted α = 0.025) and significantly lower capability scores (52.0 [48.7 to 55.3]) than general (65.1 [62.1 to 68.1]) and specialized hospitals (78.4 [67.5 to 89.2]) (*P* < 0.001 for both comparisons; adjusted α = 0.025). When looking at the ownership of facilities, public facilities had significantly lower supplies scores (49.2 [45.2 to 53.3]) than faith-based (74.4 [70.0 to 78.8]), private for-profit (65.6 [61.4 to 69.9]), and private for non-profit facilities (77.7 [72.5 to 83.0], (*P* < 0.001 for all comparisons; adjusted α = 0.025). Likewise, regarding the capabilities score, public facilities had significantly lower capabilities scores (46.0 [42.1 to 49.9]) than faith-based (65.4 [61.1 to 69.7], private for-profit (62.7 [59.1 to 66.2]), and private for non-profit facilities (73.7 [68.2 to 79.2]), (*P* < 0.001 for all comparisons; adjusted α = 0.025). In addition, private for-profit facilities had significantly lower supplies score than private for non-profit (*P* = 0.011) and faith-based facilities (*P* = 0.016); and lower capabilities score (*P* = 0.013) than private for non-profit facilities (adjusted α = 0.025).

**Fig. 2:**
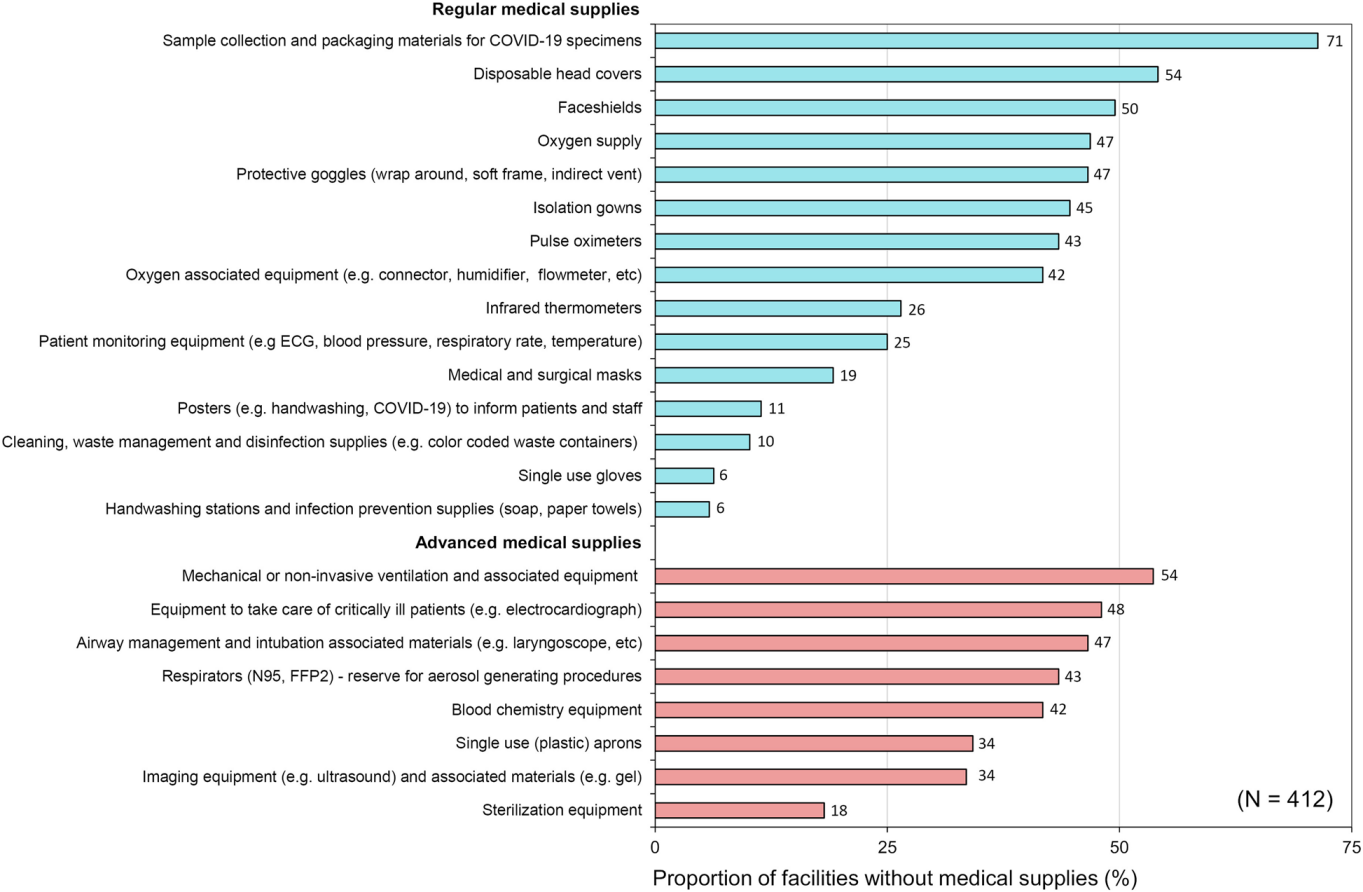
Shortages of essential medical supplies in healthcare facilities.  For the ‘Supplies score’ calculation, the SafeCare4Covid application considers as advanced medical supplies 5 items: mechanical or non-invasive ventilation equipment, equipment to take care of critically ill patients, airway management and intubation equipment, blood chemistry equipment, and imaging equipment (Supplementary Methods). However, in this figure we show 3 additional items under advanced medical supplies (N95/FFP2 respirators, single use aprons, and sterilization equipment) since these supplies were considered as not applicable (NA) to their facilities by a proportion of healthcare centres (Supplementary Table 1)

According to the SafeCare4Covid ‘Supplies checklist’ the most frequently missing commodity was sample collection and packing materials for suspected COVID-19 specimens with 71% (294/412) of facilities lacking these resources (NC) (Fig. 2, Supplementary Table 1).

In addition, oxygen supply and associated equipment were not available in 47% (193/412) and 42% (172/412) of the facilities, respectively, and pulse oximeters were missing in 179/412 (43%) healthcare centres. Medical and surgical masks (facemasks), considered the most basic PPE, were not available in 79/412 facilities (19%), from which 48% (38/79) were rural and outpatient clinics, and 43% (34/79) were of public ownership. Protective goggles, respirators (N95/FFP2) and face shields were generally not available, and single use gloves were missing in 6% (26/412) of facilities. In addition, infrared thermometers were lacking in 109/412 facilities (26%), and patient monitoring equipment, like electrocardiogram machines, tensiometers, and thermometers were absent in 103/412 (25%) healthcare centres.

**Fig. 3:**
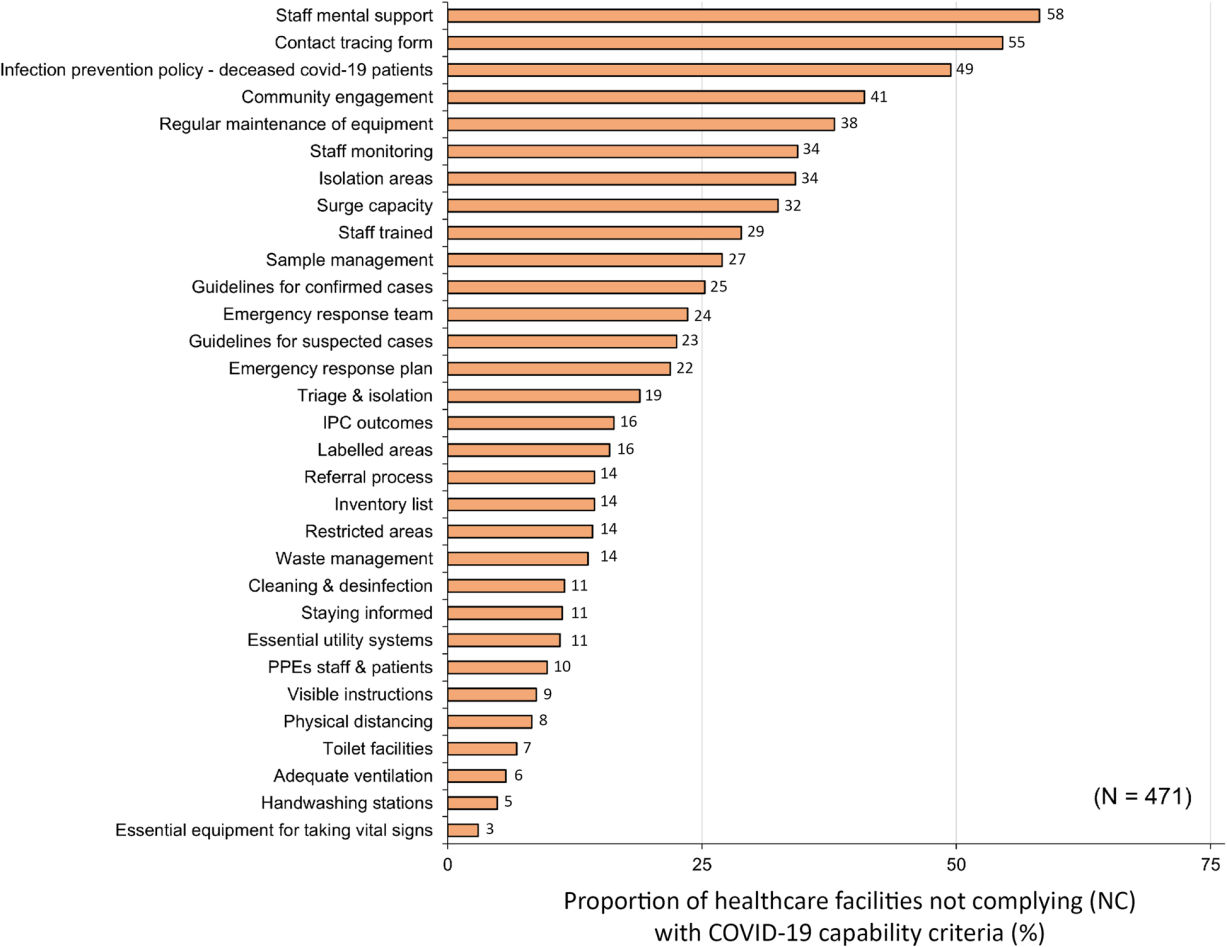
Proportion of facilities not complying with self-assessment capabilities questions (*n* = 471).  This list of capabilities corresponds to the 31 SafeCare4Covid self-assessment questions. In this figure are plotted only the proportions of facilities that were not compliant (NC) with these capability criteria in descending order

Regarding the level of preparedness against COVID-19 (31 self-assessment questions), the three most missing capability criteria (NC) among these facilities were staff mental support under ‘Capacity building’ (274/471, 58%), contact tracing forms under ‘Clinical management’ (257/471, 55%), and infection prevention policy on the management of deceased COVID-19 patients under ‘IPC’ capabilities (233/471, 49%) (Fig. 3; Tables 2 and 3).

**Table 2:** Level of compliance with ‘Clinical management’ and ‘Capacity building’ self-assessment questions

SafeCare4Covid self-assessment questions^a^ (*n* = 471)	Number of facilities per level of compliance, n (%)			
	NC	PC	FC	NA^b^
**Clinical Management (10/31 questions)**				
Do you have an emergency response plan for COVID-19?	103 (22%)	245 (52%)	123 (26%)	–
Do you have a guideline on how to triage and isolate (suspected) COVID-19 patients and is it implemented?	89 (19%)	201 (43%)	163 (35%)	18 (4%)
Do you have guidelines that describes how to manage COVID-19 suspected patients?	106 (23%)	158 (34%)	207 (44%)	–
Do you have guidelines on how to treat and manage critically ill COVID-19 patients (including those requiring ventilation)?	119 (25%)	100 (21%)	75 (16%)	177 (38%)
Does the facility have a contact tracing form for the suspected and confirmed COVID-19 cases?	257 (55%)	64 (14%)	150 (32%)	–
Do you have a risk communication and community engagement strategy?	193 (41%)	165 (35%)	113 (24%)	–
Are instructions (e.g. posters, guidelines, checklists) about COVID-19 symptoms and prevention clearly displayed to healthcare workers, patients and visitors?	41 (9%)	143 (30%)	287 (61%)	–
Do you have a process for referring COVID-19 suspected and infected patients?	68 (14%)	177 (38%)	226 (48%)	–
Do you have a protocol for the collection and referral of laboratory specimens of (suspected) COVID-19 patients?	127 (27%)	64 (14%)	146 (31%)	134 (28%)
Do you keep informed about (inter) national COVID-19 guidelines?	53 (11%)	203 (43%)	215 (46%)	–
**Capacity building (4/31 questions)**				
Do you have a COVID-19 emergency response team and are the roles and responsibilities defined?	111 (24%)	136 (29%)	159 (34%)	65 (14%)
Are all staff trained on guidelines for triaging and management of COVID-19?	136 (29%)	191 (41%)	144 (31%)	–
Are there policies and procedures for monitoring and managing healthcare professionals and support staff for COVID-19 infection?	162 (34%)	187 (40%)	122 (26%)	–
Is there mental support (counsellor) for healthcare workers (HCW) and support staff taking care of the COVID-19 patients?	274 (58%)	110 (23%)	87 (18%)	–

*Abbreviations*: *FC *full compliant, *NA *non-applicable, *NC *non-compliant, *PC *partial compliant

^a^The SafeCare4Covid application calculates the capacities score of the facilities in their preparedness against COVID-19 based on the answers to all 31 questions

^b^Facilities have the option to choose “NA” if they consider a question does not apply to their healthcare centre

**Table 3:** Level of compliance with ‘Infrastructure and supplies’ and ‘Infection prevention and control’ self-assessment questions

SafeCare4Covid self-assessment questions^a^ (*n* = 471)	Number of facilities per level of compliance, n (%)			
	NC	PC	FC	NA^b^
**Infrastructure and supplies (12/31 questions)**				–
Have you assessed and improved the surge capacity of your healthcare facility?	153 (32%)	90 (19%)	160 (34%)	68 (14%)
Is your infrastructure capable of fast-tracking patients and separating suspected COVID-19 patients from the normal patient population?	161 (34%)	124 (26%)	186 (39%)	–
Are there sufficient handwashing points including running water, liquid soap, disposable paper towels, hand sanitizer and handwashing posters available?	23 (5%)	155 (33%)	293 (62%)	–
Are there functional and clean toilet facilities and washrooms available for patients and staff?	32 (7%)	161 (34%)	278 (59%)	–
Are all the service points within the facility properly ventilated and lit?	27 (6%)	156 (33%)	288 (61%)	–
Are your service points adequately furnished for effective service provision in relation to COVID-19? (physical distance)	39 (8%)	245 (52%)	187 (40%)	–
Do all service points where suspected or confirmed COVID-19 cases are held have restricted access with proper documentation of movement in and out?	67 (14%)	113 (24%)	118 (25%)	173 (37%)
Are all service points correctly labelled and restricted access implemented at all the entry points to restricted areas (e.g. isolation ward and COVID-19 ICU)?	75 (16%)	79 (17%)	101 (21%)	216 (46%)
Do you have the required equipment (e.g. infrared thermometers, BP machines, pulse oximeters) for taking vital signs at each service point where COVID-19 patients are seen?	14 (3%)	163 (35%)	228 (48%)	66 (14%)
Do you have, maintain, and monitor an (updated) inventory list of all equipment, supplies, and medication required for COVID-19 response?	68 (14%)	143 (30%)	170 (36%)	90 (19%)
Do you have a maintenance mechanism for all essential equipment used for COVID-19 care?	179 (38%)	196 (42%)	96 (20%)	–
Do you have appropriate (back-up) arrangements for essential lifelines, like electricity and water?	52 (11%)	132 (28%)	287 (61%)	–
**Infection prevention and control (5/31 questions)**				–
Do all staff and patients have access to the required personal protective equipment (PPE) such as masks, gloves, gowns, protective glasses, and boots?	46 (10%)	265 (56%)	160 (34%)	–
Are there Infection Prevention Control (IPC), cleaning and disinfection guidelines and schedules for all the areas and equipment within the facility?	54 (11%)	152 (32%)	265 (56%)	–
Is there a documented plan for waste management at this healthcare facility?	65 (14%)	180 (38%)	226 (48%)	–
Are the outcomes for IPC defined and monitored?	77 (16%)	241 (51%)	153 (32%)	–
Is there an infection prevention policy on the management of deceased COVID-19 patients?	233 (49%)	96 (20%)	142 (30%)	–

*Abbreviations*: *FC *full compliant, *ICU *intensive care unit, *NA *non-applicable, *NC *non-compliant, *PC *partial compliant

^a^The SafeCare4Covid application calculates the capacity score of the facilities in their preparedness against covid-19 based on the answers to all 31 questions

^b^Facilities have the option to choose “NA” if they consider a question does not apply to their healthcare centre

Additionally, when looking into the ‘Clinical management’ category, guidelines for triage and isolation of suspected COVID-19 patients did not exist (89/471) or were partially implemented (201/471) in 62% (290/471) of facilities (Table 2). 264/471 facilities (56%) reported not complying (NC) with guidelines to manage suspected COVID-19 patients (106/471), or that the existing guidelines were not fully implemented.

Additionally, when looking into the ‘Clinical management’ category, guidelines for triage and isolation of suspected COVID-19 patients did not exist (89/471) or were partially implemented (201/471) in 62% (290/471) of facilities (Table 2). 264/471 facilities (56%) reported not complying (NC) with guidelines to manage suspected COVID-19 patients (106/471), or that the existing guidelines were not fully implemented (PC) (158/471) (Table 3). 41% (191/471) of facilities did not have protocols for the collection and referral of suspected COVID-19 laboratory samples (127/471), or these were not fully implemented (64/471) (Table 2). Among the ‘Infrastructure and supplies’ capabilities, the majority (243/471, 52%) of facilities did not assess their surge capacity to adapt to increases in demand expected during (COVID-19) pandemics, nor completely implemented improvements (Table 3). Fully equipped handwashing stations and functional and clean toilets were missing (178/471) or partially implemented (193/471) in 79% (371/471) of the facilities.

## Discussion

Public health emergencies, like the COVID-19 pandemic, challenge all healthcare systems, requiring an instant reorganization of clinics and hospitals. In such circumstances there is little time to reflect on where to allocate the economic and human resources to address the current emergency while continuing to provide basic healthcare services to the population, guarantying the safety of both patients and health workers [34]. The skillset of quality improvement (QI) people can be of great help and showed to be essential for the successful assessment of COVID-19 epidemic preparedness and implementation of required changes in many healthcare centres worldwide. Here, we show the results of a survey study of the level of preparedness of healthcare facilities to COVID-19 with SafeCare4Covid, a tool using a QI methodology that was rapidly adapted to COVID-19 care and management by QI professionals with extended experience in QI implementations in Africa.

### Main findings

This study is to our knowledge the first multi-country comprehensive study in Africa that systematically collects information on (COVID-19) epidemic preparedness from healthcare centres using a QI methodology. We assessed 471 healthcare facilities from 11 African countries from May–November 2020, from which 66.5% (313/471) were part of the SafeCare programme. All in all, we quantified alarming gaps in critical COVID-19 preparedness capabilities and lack of life-saving PPEs. Primary healthcare centres, facilities located in rural areas and of public ownership were the less prepared, and clearly left behind regarding epidemic preparedness efforts.

First, the SafeCare4Covid ‘Clinical Management’ and ‘IPC’ components evaluated facility readiness to attend COVID-19 patients. In general, emergency response plans and teams should be pre-developed and implemented as soon as a major public health emergency takes place. Regarding COVID-19, we assessed processes that address the capacity of the facilities against a set of requirements to: effectively managing risks; identify gaps in the facility’s readiness in terms of knowledge and infrastructure/medical supplies required to take care of COVID-19 patients; swift adaptation to increased demands ensuring the continuation of essential services (business continuity); clear and accurate internal and external communication; the effective use of scarce resources; and a safe environment for healthcare workers and patients [35].

Within the ‘Clinical management’ component, we found that an alarming number of facilities reported no or partial compliance (NC or PC) with having an emergency response plan and emergency response team (Fig. 2; Table 2). These findings corroborate gaps we observed on critical COVID-19 pandemic capabilities, such as triage and isolation of suspected COVID-19 patients that were NC or PC in two thirds of facilities. Of concern, some facilities even considered triage and isolation capabilities not to apply (NA) to their centres (Table 2), probably due to a lack of understanding of their needs and implementations in a facility of their size and scope. Obstacles to comply with this capability were related to infrastructure limitations (NC or PC) to separate suspected COVID-19 cases from the regular patient population (Fig. 2,  Table 3). A solution to the lack of space could be installing outdoor triage stations for the assessment of patients with respiratory symptoms [36]. Tents with adequate ventilation can be an option whenever weather hinders outdoor screenings. Same solutions (outdoor stations) can be implemented for the isolation of suspected cases. Importantly, outdoors triage areas not only help to improve readiness of the healthcare facilities to attend COVID-19 patients, but at the same time bring a visible message of safety to the community and patients accessing the facility with other medical conditions. However, they could also generate fears. Therefore, these measures should be accompanied by community engagement to prevent this effect. In addition to triage and isolation procedures, there should be the possibility to perform or refer for COVID-19 diagnostic tests of suspected patients. This option was missing or not fully implemented in more than half of facilities (Table 2, Supplementary Table 1). This finding agrees with what has been stated by several stakeholders about the limited diagnostic capacity of COVID-19 in Africa [5, 13, 14, 37], and therefore the potential underreporting of cases.

Furthermore, an alarming number of facilities (~ 45%) reported that oxygen, oxygen supply-associated equipment, and pulse oximeters were not available (Fig. 1, Supplementary Table 1). Moderate, severe, and critically ill COVID-19 patients require oxygen to treat the hypoxemia induced by respiratory illness. The struggle of increased demand for oxygen during the COVID-19 pandemic has been internationally recognized by the ‘COVID-19 Oxygen Emergency Taskforce’ established by the Wellcome Trust, Unitaid, and WHO. These agencies reported at the time of the pandemic that “more than half a million patients with COVID-19 in LMICs need oxygen every day and shortages are causing preventable deaths” [38]. Moreover, these estimates do not include the impact of oxygen shortages for new-borns, children with pneumonia and/or malaria, and other patients requiring oxygen therapy [38]. These statements align with our findings and urge the need to prioritize oxygen in budgets and strategy.

In addition, we found generally deficient official COVID-19-related communications between the local health authorities and the healthcare centres, and between the latter and the community. This is revealed by the many healthcare facilities that we found were left uninformed about how to manage suspected COVID-19 patients, or what process to follow to refer a suspected/confirmed case to other facilities (Table 2). Additionally, we observed lack of engagement with the community (Table 2) that could lead to fears, healthcare facility-avoiding behaviours, and deficient/inappropriate use of PPEs [39]. Direct, fast, and concise communications are greatly needed in emergency times. In settings as Africa where many healthcare centres are located in remote areas, mobile digital technologies could bring reliable, up-to-date information in real-time to healthcare professionals and the community.

Regarding ‘IPC’ capabilities, we found serious breaches of COVID-19 preparedness in the majority of participating facilities. Healthcare workers in contact with or taking care of COVID-19 patients are at higher risk of infection than the general population [12, 40]. Compared with SARS-CoV and H1N5 viruses, SARS-CoV-2 is much more efficient in infecting human conjunctiva and upper respiratory airways [41]. It also has longer incubation periods, with significant pre-symptomatic virus shedding, complicating the control of this infection when compared with previous coronavirus epidemics [41]. Finally, SARS-CoV-2 can replicate in colorectal cells, implicating a possible faecal-oral route of transmission, when insufficient IPC measures are applied. Therefore, to prevent facility-acquired infections in healthcare personnel and non-COVID-19 patients, the WHO recommends the use of appropriate PPE, hand hygiene best practices, implementation of universal masking policies, and adequate IPC training and education [10]. Of important concern, in our study two third of facilities reported lack or insufficient access to required PPEs (Table 3). In line with this, many facilities reported not having medical masks, respirators N95/FFP2, protective goggles, and face shields (Fig. 1, Supplementary Table 1). It is recommended that healthcare workers (HCWs) wear respirators or a well-fitting medical mask and eye protection during patient care encounters [36]. Due to the scarcity of respirators N95/FFP2 in Africa, it is better to reserve these PPEs for aerosol-generating procedures. This is a lesson learned for future epidemics, underscoring the need of rationalization of limited PPEs and local production of such supplies. Extended use of eye protection (referring to the use of same eye protection for different contact encounters) and disinfection and reuse of goggles and face shields are advised to cope with shortages [36]. In these circumstances, goggles and face shields should be dedicated to one HCW and discharged when damaged [36]. The use of cloth masks is a solution for patients attending the facilities when having limited access to medical masks and should be washed after attending a healthcare centre. Importantly, in agreement with previous findings in Tanzanian in 2018 [11], many centres did not work with IPC, cleaning and disinfection guidelines, nor had defined or monitored IPC outcomes (Table 3). Although the likelihood of infection with COVID-19 while handling human remains is low [42], more than two third of facilities scored mostly NC or PC with IPC policies on the management of deceased COVID-19 patients (Fig. 2,  Table 3).

Due to its fragile healthcare systems, the COVID-19 pandemic represented a great burden for the continent, similarly to the impact of the 2014–2016 Ebola Virus Disease epidemic on West Africa [43, 44]. For COVID-19 it has been estimated that HIV-, tuberculosis-, and malaria-related deaths over 5 years may be increased by up to 10%, 20% and 36%, respectively [45]. Some indications of the accuracy of these predictions have been recently found in some African studies [7, 46, 47]. Importantly, basic healthcare services as child vaccinations and antenatal care were also compromised [16, 21], due to lack of offer (overpassed capacity) or demand (change in care-seeking behaviour) of these services. The maintenance of such essential medical services is of paramount importance in Africa, while having the capacity to cope with the increase demand of services. In this regard, only 34% of the facilities here studied were fully compliant with assessing and improving their surge capacity (Table 3). The latter is important to guarantee the safety of non-COVID-19 patients, and simultaneously prevent the collapse of the healthcare system and bankrupt of private healthcare centres.

Finally, SafeCare4Covid evaluated aspects related to human resources. The pandemic put an unprecedented strain on healthcare systems and HCWs globally. Hiring new personnel was often not possible because of scarcity, stigma, fear, and insufficient financial resources in LMICs. In addition, many employers requested HCWs with risk factors (e.g. older, with underlying conditions) to stay at home, reducing even more the work force in healthcare facilities. This has led to a catastrophic shortage of medical professionals in most African countries [14]. Hence, frontline, and non-frontline HCWs suffered from physical and mental exhaustion, with high rates of burnout, insomnia, depression, and anxiety [48–50]. Unfortunately, some suicide cases have been reported among doctors worldwide [51]. In order to prevent this, healthcare personnel need to gain confidence and feel safe when taking care of COVID-19 patients, or even when working in another unit of the healthcare centre during an outbreak. Training in the management of COVID-19 patients and establishing an emergency response team can help relieve these worries. We found these two capabilities missing in a quarter of facilities (Table 2). Most importantly, regular monitoring of the health status of HCWs with frequent COVID-19 testing and mental support are greatly needed. In reality, around 60% of facilities did not have mental health support for healthcare personnel, and a third had no procedures in place to closely monitor HCWs (Fig. 2,  Table 2). To provide mental health support for HCW amidst a pandemic is challenging but necessary. In this regard Africa could bring COVID-19-related mental support to HCWs and the community throughout experienced counsellors for people living with HIV. Many of these professionals provided COVID-19 counselling in Kenya, bringing up-to-day information to HCWs and the community, and reducing fears in people that do not want to be tested due to stigma and ideas of possible hospitalizations they cannot afford (unpublished data).

### Limitations and strengths of the study

This study has some limitations. First, SafeCare4Covid is a self-assessment tool. We were able to verify the information collected only from a small number of facilities where PharmAccess is actively engaged with through projects after this study period. In Kisumu, Kenya we found that some facilities had 20-point lower scores in the onsite assessment compared to the self-assessment (range, 0‒100), mainly due to difficulties differentiating between ‘partially compliant’ and ‘fully compliant’ criteria [30]. Currently the SafeCare methodology continue with self-assessments to incentivise QI before onsite assessments, perform broad quality mapping, or assess quality in remote and fragile healthcare regions. Uploading pictures of relevant QI activities is being implemented to help the QI teams confirm or correct the self-assessment scores remotely. Moreover, self-assessment frameworks are regularly used by international health authorities such as the WHO to identify key issues requiring attention and improvement of their healthcare programs [52] and in times of emergency are likely the fastest and most feasible option. Second, participation in the tool is likely to be biased, since 66.5% of facilities in this study were actively approached because they were already participating in the existing SafeCare programme, and others through local partners that PharmAccess works with. Therefore, it is not a randomized sample and we do not claim that these results represent all African healthcare facilities. Third, considering that the use of the App was voluntary, probably these facilities represent a group of healthcare centres willing to improve their preparedness, and therefore that the overall situation may be even more dismal in Africa. Fourth, these facilities did not repeat the assessments, hence this analysis refers to the baseline scores only, and it is possible these changed with time. In another group of facilities, we noticed repeated assessments were done only when requested. Identified barriers were staff turnover and the challenge of prioritizing self-assessments over the difficulties of keeping the facility business afloat during the pandemic [30]. Lastly, under- or over-reporting was unlikely since there was no financial incentive on the score of the self-assessment, but rather learning and capacity building was promoted as the main purpose of using the App. Nevertheless, many of the results of our study are coherent with the local reality, e.g. that rural facilities have less capabilities and are less equipped than urban facilities [53, 54]; and also regarding the lack of PPEs [13, 14, 50], oxygen supply [38], and diagnostic capacity [5, 13, 14, 37]. This and other findings support the validity of our data and underscore the important role of digital applications in times of emergency.

### Implications for stakeholders and policymakers

Surveillance is key to obtain and have access to epidemic preparedness metrics [29]. Digital tools can help identifying important gaps of healthcare facilities in Africa and provide solutions adapted to the local needs. Due to the ubiquity of mobile phones in Africa, such digital self-assessment tools provide the opportunity of a paradigm shift from the centralized approach of fighting epidemics in sub-Saharan (SSA) to one complemented with decentralized self-assessment tools [55]. The latter would allow for a quick and low-cost inclusion of healthcare providers that are difficult to reach during lockdowns because they are located in remote, rural regions, not only to assess their preparedness but also bringing official information about the ongoing epidemic to combat misinformation [30]. Simultaneously, gathered data can be used by local stakeholders to inform targeted interventions in real time. For instance, here we show that PPE shortages and COVID-19 preparedness gaps affected African healthcare facilities unevenly, leaving rural facilities, outpatient clinics, and public and private for-profit healthcare centres at the greatest disadvantage. In a continent in which 60% of the population live in rural areas [56], 85% of healthcare facilities are primary healthcare centres (outpatient clinics) [57], and at least 50% percent of healthcare services are provided by the private sector [58, 59], remote digital mapping can greatly support stakeholders and policymakers to assess the fundamental needs/gaps in epidemic preparedness of these facilities. National responses during COVID-19 were focussed on tertiary hospitals, although the real fight was taking place at the primary healthcare facilities where the majority of Africans seek their care. We found that gaps are the most prominent and impactful particularly in these healthcare facilities. Primary healthcare and epidemic preparedness and response are historically isolated sectors [29] that need to be united to build resilient healthcare systems through data-insights. A good example of the latter is the last Sudan Virus Diseases (Ebola) outbreak in Uganda which index case was treated in a regional referral hospital but whom place of residence was a village in a subcounty, where it could have been detected earlier in a primary healthcare centre [60]. Nevertheless, national surveillance and contact tracing were immediately implemented at a community level and the outbreak was contained and an epidemic was prevented. Hence, coordinated work between primary healthcare centres and regional referral hospitals is necessary since both are very important. Importantly, in our study many of the required remedies to the deficient COVID-19 preparedness we found are of relatively low cost, but need to be decentralized, such as the procurement and distribution of PPEs and other commodities. Hence, data collected in real-time and shared with key stakeholders can help prioritizing where to allocate (limited) supplies in times of severe shortages, but also where to allocate patients when hospitals are overflowing. An evidence-based response has been barely possible in most African countries, as data about the capabilities of public and the private healthcare facilities to manage the pandemic, as well as to protect non-COVID-19 patients, were scarce. Lastly, if healthcare facilities are transparent when self-reporting on availability of supplies and PPEs, and measures can be put in place to prevent inaccurate reporting, digital tools can help ensure that national and international funding are distributed accountably and transparently at the place they are needed. We actively engaged public and private stakeholders during the COVID-19 pandemic sharing real-time data though the SafeCare4Covid dashboard to advocate for support of facilities in SSA to improve their preparedness, with some successful projects (Please see ‘Dissemination plans’). In Kenya a group of facilities (*n* = 67) that assessed their COVID-19 preparedness with the SafeCare4Covid tool received technical assistant tailored to their COVID-19 preparedness needs in 2021 by the SafeCare and MCF programmes. Follow-up self-assessments were performed upon request (not spontaneously) in January 2022 showing significant improvements in capabilities scores from baseline (mean 60.1 [95% CI 54.9 to 65.3]) to follow-up (69.9 [66.0 to 73.8]) (*P* = 0.001, paired t test). For future epidemics, adopting self-assessment tools at a network level would allow for similar tailored training and planned follow-up assessments, enhancing the impact of such digital interventions on QI and epidemic preparedness [30].

## Conclusions

A large number of facilities in our study miss critical COVID-19 preparedness capabilities, including lack of diagnostic capacity, with many HCWs fighting the pandemic without proper PPEs putting communities at risk. The facilities at greatest need are the very ones providing healthcare to the majority of patients: primary healthcare centres located in rural areas of public and private-for-profit ownership. Aggregate time-tagged and geo-marked data provide valuable information and help policy makers and health managers to take data-informed decisions. We plea the local governments, Africa CDC, COVAX and other initiatives for maintaining a basic and decentralized digital health ecosystem involving primary healthcare centres in rural settings. This would allow for rapid scaling up during an emergency and provide valuable real-time data-insights to react to and monitor outbreaks and build resilient healthcare systems.

### Supplementary Information

**Supplementary Material 1.**

**Supplementary Material 2.**

## Data Availability

All data from this analysis can be shared through dashboards or other forms upon a data sharing and confidentiality agreement between both parties. The lead authors affirm that the manuscript is an honest, accurate, and transparent account of the study being reported; that no important aspects of the study have been omitted.

## Abbreviations

COVID-19: The coronavirus disease 2019
SARS-CoV-2: Severe acute respiratory syndrome coronavirus 2
IPC: Infection prevention and control
PPE: Personal protective equipment
WHO: World Health Organization
EIDs: Emerging infectious diseases
LMICs: Low-middle income countries
QI: Quality improvement
QIP: Quality improvement plan
NC: Non-compliant
PC: Partial compliant
FC: Fully compliant
NA: Not applicable
HCWs: Healthcare workers
SSA: Sub-Saharan Africa
