# Supplementary material for: Preparing healthcare facilities in sub-Saharan Africa for future outbreaks: insights from a multi-country digital self-assessment of COVID-19 preparedness
Source: BMC Health Serv Res. 2024 Feb 28;24:254. doi: 10.1186/s12913-024-10761-2 (PMC10900561; doi:10.1186/s12913-024-10761-2)
Supplement: Supplementary file 1 — Supplementary Material 1. [file 12913_2024_10761_MOESM1_ESM.pdf]

Supplementary Table 1. Verification list of 23 medical supplies

Checklist of medical supplies[a] (n = 412)	Number of facilities with/without supplies, n (%)		
	Yes	No	NA[b]
Sample collection and packaging materials for COVID-19 specimens	118 (29%)	294 (71%)	–
Disposable head covers	189 (46%)	223 (54%)	–
Face shields (single use)	208 (50%)	204 (50%)	–
Oxygen supply	219 (53%)	193 (47%)	–
Protective goggles (wrap around, soft frame, indirect vent)	220 (53%)	192 (47%)	–
Isolation gowns	228 (55%)	184 (45%)	–
Pulse oximeters	233 (57%)	179 (43%)	–
Oxygen associated equipment (e.g. connector, humidifier, tubes, flowmeter, nasal oxygen canula, with prongs, masks, etc)	240 (58%)	172 (42%)	–
Infrared thermometers	303 (74%)	109 (26%)	–
Patient monitoring equipment (e.g ECG, blood pressure, respiratory rate, temperature)	309 (75%)	103 (25%)	–
Medical and surgical masks	333 (81%)	79 (19%)	–
Posters (e.g. handwashing, COVID-19) to inform patients and staff	365 (89%)	47 (11%)	–

Cleaning, waste management and disinfection supplies (e.g. color coded waste containers and appropriate waste bags, disinfectants, disinfection chemicals, brooms, rugs and other cleaning tools)	370 (90%)	42 (10%)	–
Single use gloves	386 (94%)	26 (6%)	–
Handwashing stations and infection prevention supplies (soap, paper towels, alcohol-based hand sanitizer, cough tissues, closed trash cans, etc.)	388 (94%)	24 (6%)	–
Mechanical or non-invasive ventilation and associated equipment for adult, paediatric and neonates - mechanical ventilation requires trained staff	72 (17%)	221 (54%)	119 (29%)
Equipment to take care of critically ill patients (e.g. electrocardiograph, defibrillator(s), syringe pumps, suction pumps, IV infusion, medication, etc)	119 (29%)	198 (48%)	95 (23%)
Airway management and intubation equipment and associated materials (e.g. laryngoscope, end tidal CO2 detector, endotracheal tubes, etc)	108 (26%)	192 (47%)	112 (27%)
Respirators (N95, FFP2) - reserve for aerosol generating procedures	195 (47%)	179 (43%)	38 (9%)
Blood chemistry equipment	158 (38%)	172 (42%)	82 (20%)
Single use (plastic) aprons	239 (58%)	141 (34%)	32 (8%)
Imaging equipment (e.g. ultrasound) and associated materials (e.g. gel)	203 (49%)	138 (34%)	71 (17%)

Sterilization equipment	311 (75%)	75 (18%)	26 (6%)

[a]The SafeCare4Covid application calculates the supplies score of the facilities based on the answers to these 23 verification list questions.

[b]Facilities have the option to choose “NA” if they consider a supply does not apply to the level/size of their healthcare center.

Abbreviations: NA, non applicable.
