# Supplementary material for: Preparing healthcare facilities in sub-Saharan Africa for future outbreaks: insights from a multi-country digital self-assessment of COVID-19 preparedness
Source: BMC Health Serv Res. 2024 Feb 28;24:254. doi: 10.1186/s12913-024-10761-2 (PMC10900561; doi:10.1186/s12913-024-10761-2)
Supplement: Supplementary file 2 — Supplementary Material 2. [file 12913_2024_10761_MOESM2_ESM.pdf]

Supplementary Methods

Preparing Healthcare Facilities in Sub-Saharan Africa for Future Outbreaks: Insights From a Multi-Country Digital Self-Assessment of COVID-19 Preparedness

Gloria P. Gómez-Pérez[1,2*], Aafke E. de Graaff[1], John T. Dekker[1], Bonifacia B. Agyei[3], Ibironke Dada[4], Emmanuel Milimo[5], Marilyn S. Ommeh[5], Peter Risha[6], Tobias F. Rinke de Wit[1,2], Nicole Spieker[1]

1 PharmAccess Foundation, Amsterdam, The Netherlands

2 Amsterdam Institute for Global Health and Development, University of Amsterdam, Amsterdam, The Netherlands

3 PharmAccess Ghana, Accra, Ghana

4 PharmAccess Nigeria, Lagos, Nigeria

5 PharmAccess Kenya, Nairobi, Kenya

6 PharmAccess Tanzania, Dar es Salaam, Tanzania

*Corresponding author: Gloria P. Gómez-Pérez (email: p.gomez@pharmaccess.org)

SafeCare4Covid score calculations

1. Capabilities score

There are in total 31 capabilities measured.

The formula of the capability score is as follows:

- *Capabilities score* = (# fully compliant [FC] capabilities criteria)(100/(31 - # not applicable [NA] capabilities criteria)) + (# partially compliant [PC] capabilities criteria)(50/(31 - # NA capabilities criteria))

Not compliant (NC) capabilities = not used for calculation.

Example: A facility “X” that has 10 FC capabilities, 10 PC capabilities, 5 NC capabilities, and 6 NA capabilities will have the following calculation for the capabilities score:

- *Capabilities score facility “X”* = (10)(100/(31 – 6)) + (10)(50/(31 – 6)) = (10)(100/25) + (10)(50/25) = (10)(4) + (10)(2) = 40 + 20 = 60.

2. Supplies score

In the supplies checklist there are in total 23 essential medical supplies measured (Supplementary Table 1):

8 personal protective equipment (PPEs): disposable head covers, faceshields, protective goggles, isolation gowns, medical and surgical masks, single use gloves, N95/FFP2 respirators, and single use (plastic) aprons.

5 monitoring/oxygen supplies: oxygen supply, oxygen associated equipment, pulse oximeters, infrared thermometers, and patient monitoring equipment.

5 advanced supplies: imaging equipment, blood chemistry equipment, airway management and intubation equipment, mechanical or non-invasive ventilation equipment, and equipment to take care of critically ill patients.

5 infection prevention supplies: sample collection and packaging materials for covid-19 specimens, posters to inform patients and staff, cleaning, waste management and disinfection supplies, handwashing stations, and sterilization equipment.

The formula of the supplies score is as follows:

- PPEs score = (# Yes / (8 – # NA)) 100
- Monitoring/oxygen supplies score = (# Yes / (5 – # NA)) 100
- Advanced supplies score = (# Yes / (5 – # NA)) 100
- Infection prevention supplies score = (# Yes / (5 – # NA)) 100

Total supplies score = ((PPR score) + (monitoring/oxygen score) + (advanced supplies score) + (infection prevention supplies score))/4

Example: A facility "Y" that has:

- PPE score = 5 PPEs (yes) + 1 NA PPE + 2 missing PPEs (no) = (5/(8 – 1)) 100 = (5/7) 100 = (0.71) 100 = 71
- Monitoring/oxygen supplies score = 4 supplies (yes) + 1 NA supply + 0 missing supplies (no) = (4/5 -1) 100 = (1) 100 = 100
- Advanced supplies score = 4 supplies (yes) + 1 NA supply + 0 missing supplies (no) = (4/5 -1) 100 = (1) 100 = 100
- Infection prevention supplies score 4 supplies (yes) + 1 NA supply + 0 missing supplies (no) = (4/5 -1) 100 = (1) 100 = 100

Supplies score facility "Y" = (71 + 100 + 100 + 100)/4 = (371)/4 = 92.75.
